# Erratum to “Effects of colchicine on SAH-induced vascular damage in an experimental subarachnoid hemorrhage model” [Turkish Journal of Medical Sciences 56 (2) 2026 597–607]

**DOI:** 10.55730/1300-0144.6379

**Published:** 2025-07-26

**Authors:** Emine DEMİR, Cumaali DEMİRTAŞ, Cansu SÖNMEZ, Eray Metin GÜLER, Sarper KOCAOĞLU, Hakan BEYAZTAŞ, Eyüp ÇETİN

**Affiliations:** 1 Department of Neurosurgery, Ministry of Health Üsküdar State Hospital, İstanbul, Turkiye; 2 Department of Physiology, University of Health Sciences, Hamidiye Health Sciences Institute, İstanbul, Turkiye; 3 Department of Pathology, University of Health Sciences, Haydarpaşa Numune Training and Research Hospital, İstanbul, Turkiye; 4 Department of Medical Biochemistry, University of Health Sciences, Hamidiye Medical Faculty, İstanbul Turkiye; 5 Department of Neurosurgery, Health Sciences University Haydarpaşa Numune Training and Research Hospital, İstanbul, Turkiye; 6 Department of Biochemistry, Health Sciences University Hamidiye Medical Faculty, İstanbul, Turkiye; 7 Department of Neurosurgery, Health Sciences University Haydarpaşa Numune Training and Research Hospital, İstanbul, Turkiye

**Keywords:** Subarachnoid haemorrhage, colchicine, oxidative stress, inflammation, cerebrovascular circulation

## Abstract

**Background/aim:**

Subarachnoid haemorrhage (SAH) is a severe cerebrovascular condition with high morbidity and mortality. Inflammation and oxidative stress are key factors in its pathophysiology, particularly in secondary vascular injury. This study aimed to evaluate the effects of colchicine on vascular integrity and biochemical markers following SAH in an experimental rat model.

**Materials and methods:**

Eighteen female Sprague-Dawley rats were divided into three groups: Control (no SAH), SAH (induced without treatment), and SAH + Colchicine (1 mg/kg intraperitoneally at 1 h and 24 h). SAH was induced by autologous blood injection into the cisterna magna. Rats were sacrificed 48 h post-induction. Serum and tissue samples were analyzed for oxidative stress indices and inflammatory cytokines, and histopathological evaluations were performed to assess vascular injury.

**Results:**

SAH caused significant increases in oxidative stress and pro-inflammatory cytokines compared to the Control group. Tissue TOS and OSI levels were higher, while TAS was lower in both SAH and colchicine groups (p < 0.05). Serum cytokines (IL-1β, IL-6, TNF-α) and OSI were highest in the colchicine group, intermediate in SAH, and lowest in Control, whereas TAS showed the opposite pattern (p < 0.05). Thiol-disulfide homeostasis shifted toward oxidation, with reduced total/native thiols and elevated disulfide ratios. Histologically, colchicine partially reduced endothelial damage and inflammation, but basilar artery diameter and wall thickness remained unchanged.

**Conclusion:**

At the administered dose and 48-hour time point, colchicine produced partial histological improvement without reducing systemic oxidative or inflammatory markers. Some serum indices were higher than in untreated SAH, suggesting a limited short-term protective effect. Further studies are required to explore optimal dosing and timing for colchicine as a potential neuroprotective agent in SAH-related vascular injury.

## 1. Introduction

SAH is one of the most acute and severe subtypes of stroke, characterized by high morbidity and mortality rates [1]. Inflammation is considered a major cause of vasospasm following SAH, with inflammatory markers becoming evident as early as three hours posthaemorrhage. Inflammatory cells, including leukocytes, granulocytes, monocytes, and lymphocytes, migrate to the affected area, releasing pro-inflammatory cytokines such as Tumour Necrosis Factor Alpha (TNF-α), Interleukin-1 Beta (IL-1β), Interleukin-6 (IL-6), and IL-8, thereby initiating the acute phase of inflammation. Among these cytokines, IL-6 affects vascular endothelial cells by increasing endothelial permeability [2].

Despite numerous studies on cerebral vasospasm following SAH, the underlying pathogenic mechanisms remain unclear. Blood clots in the subarachnoid space, arterial damage, intracranial hypertension, cerebral ischemia, or arterial spasm are potential contributors, though the exact processes remain uncertain. While arterial spasm is believed to be complex and multifactorial, inflammation triggered by blood in the subarachnoid space is considered the primary cause of cerebral vasospasm [3]. Studies have shown elevated IL-6 levels within the first week after SAH [4].

Endothelin-1 (ET-1), one of the most potent vasoconstrictors, has been closely linked to the development of vasospasm in SAH. Increased ET-1 levels in cerebrospinal fluid (CSF) and plasma, along with elevated levels of acute-phase reactants such as IL-1β, IL-6, and TNF-α, correlate strongly with vasospasm development [5]. Following SAH, inflammatory cytokines such as IL-1β, IL-6, and TNF-α reach peak levels in the CSF due to tissue damage. In vitro studies have demonstrated that these cytokines can directly pass into the subarachnoid space, cross the adventitial surface, and be taken up by smooth muscle cells in the basal arteries, contributing to vasospasm and increasing vascular tone [6].

Early implementation of anti-inflammatory or anti-cytokine strategies has been suggested as a potential approach to prevent the hemodynamic complications associated with SAH [7]. After SAH-induced endothelial damage, platelet adhesion to the endothelium leads to platelet aggregation and thromboxane-A2 (TX-A2) synthesis. Additionally, free radicals produced post-SAH activate phospholipases, releasing fatty acids like arachidonic acid from cell membranes. This process results in the formation of leukotrienes, thromboxane, and prostaglandins, with TX-A2 acting as a potent vasoconstrictor [8].

Colchicine has been shown to reduce neutrophil migration to sites of tissue damage by binding to microtubules and limiting neutrophil elasticity, thereby preventing their spread into tissues [9]. Studies have also demonstrated that colchicine suppresses TNF-α production by macrophages and decreases TNF-α receptor expression on endothelial cells [10]. Recent studies using low-dose colchicine (0.5–1.0 mg/day) have demonstrated its therapeutic potential in cardiovascular diseases, including coronary artery disease, postoperative atrial fibrillation, cardiac hypertrophy-related heart failure, and similar conditions. Its beneficial effects have been attributed to the inhibition of inflammasome activation, suppression of leukocyte and IL-1β production, and reduction of neutrophil activation and degranulation, ultimately improving endothelial function [11–13].

Animal studies have shown that colchicine improved left ventricular function and reduced NLRP3 inflammasome activity in myocarditis caused by Coxsackievirus B3 (CVB3) infection [14]. Furthermore, colchicine has been found to reduce cytokines implicated in vasospasm following SAH, including TNF-α, IL-1β, IFN-γ, IL-18, and IL-6, as well as to suppress leukocyte functions [15].

Additionally, studies involving diabetic patients undergoing stent placement or coronary artery bypass grafting have reported that colchicine administration before revascularization reduced infarct size and ischemia-reperfusion injury. Based on these findings, colchicine is thought to have potential beneficial effects on infarct size and ischemia in patients experiencing SAH [16–21].

Although numerous treatment recommendations for cerebral vasospasm following SAH have been proposed in clinical guidelines and research studies, challenges remain in identifying a definitive and effective treatment method. Current vasospasm therapies primarily focus on alleviating arterial narrowing through mechanical and pharmacological interventions, despite the unclear underlying causes and mechanisms of vasospasm. Given colchicine’s antiinflammatory properties and its mechanism of action on the endothelium, we believe that exploring its potential to prevent vasospasm could yield meaningful results.

A review of the existing literature reveals that while various pharmacological agents have been tested in experimental models of SAH, no studies involving colchicine have been conducted. In this regard, our research represents a novel approach, contributing a unique perspective to the current body of knowledge.

## 2. Materials and methods

### 2.1. Statistical Methodology

Mean, standard deviation, median, minimum, maximum value frequency and percentage were used for descriptive statistics. The distribution of variables is measured by Kolmogorov Smirnov and Shapiro-Wilk tests. ANOVA (Tukey test) and independent samples t test were used to analyse independent quantitative data with normal distribution. Kruskal-Wallis and Mann-Whitney U tests were used in the analysis of independent quantitative data with nonnormal distribution. SPSS 27.0 was used for statistical analyses.

### 2.2. Experimental Groups

The study used 18 female Sprague Dawley rats weighing 200–250 grams, divided into three experimental groups of 6 rats each:

1. Control Group: No SAH induction. Rats were sacrificed to demonstrate normal basilar artery anatomy.
2. SAH Group: Rats with induced SAH but no colchicine treatment, sacrificed on day 2 (48 hours post-SAH).
3. SAH + Colchicine Group: Rats with induced SAH receiving 1 mg/kg intraperitoneal colchicine at 1 hour and 24 hours post-SAH, sacrificed on day 2 (48 hours post-SAH).

### 2.3. SAH Model Induction

The rats were fasted for 12 hours before the procedure. General anaesthesia was induced using Ketamine Hydrochloride (50 mg/kg, Ketalar vial, Eczacıbaşı İlaç Sanayi, Istanbul) and Xylazine (10 mg/kg, Rhompun injectable vial, Bayer Türk Kimya Sanayi, Istanbul) via intraperitoneal injection.

To create the SAH model, a 30G needle was inserted percutaneously into the cisterna magna to withdraw 0.2 ml of CSF, followed by injecting 0.2 ml of autologous blood from the femoral artery. This established a single-haemorrhage model. Clinical assessments for vasospasm were performed using the Bederson and Garcia scoring systems until the rats were sacrificed. The colchicine dose of 1 mg/kg was selected based on prior studies demonstrating its antiinflammatory and neuroprotective effects in preclinical models of cerebrovascular injury. Specifically, similar doses have been shown to reduce inflammatory cytokine levels and mitigate oxidative stress in experimental models of central nervous system inflammation. This dose also aligns with established pharmacological protocols, ensuring effective tissue penetration while minimizing potential toxicity [14].

### 2.4. Sacrificing and Tissue Processing

Rats were sacrificed under general anaesthesia via cervical dislocation. Approximately 3 ml of intracardiac blood was collected into serum gel tubes containing clot activators before sacrifice. The samples were centrifuged at 3000 x g for 10 minutes to separate the serum, which was stored at −80°C until biochemical analysis. Serum samples and tissue supernatants were analysed for biochemical markers. Tissue-derived parameters were normalized based on total protein concentrations measured using Bradford protein assay.

A front parietooccipital craniectomy was performed to extract intracranial structures extending to the foramen magnum while preserving anatomical integrity. The samples were fixed in 10% buffered formaldehyde for 24 hours.

The brainstem and basilar artery sections were processed, embedded in paraffin blocks, and sectioned at 5-micrometer thickness. Haematoxylin-eosin staining was performed, and sections were examined under a light microscope by two blinded observers. Basilar artery lumen area and arterial wall thickness were measured. Group comparisons were performed using the appropriate test according to distribution (ANOVA or Kruskal–Wallis).

## 3. Results

### 3.1. Histopathological Analysis

Histopathological evaluation was performed to assess the structural and inflammatory changes in the basilar artery across experimental groups. Representative images from each group are included to demonstrate normal anatomy, SAH-induced pathological changes, and the effects of colchicine treatment.

The control group demonstrated a normal histological structure of the basilar artery, as shown in Figure 1. The arterial lumen was unobstructed, maintaining a wide and open passage for blood flow, with a smooth and intact endothelial lining. The arterial wall exhibited a uniform thickness, ensuring consistent structural integrity. There were no signs of endothelial damage, inflammation, or vascular remodelling, indicating a healthy and unstressed circulatory environment. The layers of the arterial wall, including the tunica intima, media, and adventitia, were clearly distinguishable, demonstrating their well-preserved architecture. The absence of leukocyte infiltration and apoptotic cells further emphasizes the lack of pathological changes. This image serves as a crucial reference for comparing the control group’s normal vascular structure with the pathological changes observed in the SAH and colchicine-treated groups. By establishing this baseline, any structural disruptions, inflammatory responses, or vascular remodelling in the experimental groups can be more accurately identified and analysed. The lack of endothelial dysfunction or thickening highlights the physiological state of the basilar artery in healthy conditions, where normal cerebrovascular function is maintained without external stressors such as haemorrhage or drug intervention.

Figure 2 represents the basilar artery in the SAH group, showing representative the histopathological changes associated with SAH. Unlike the control group, the arterial lumen in the SAH group appears more constricted, likely due to vasospasm and inflammatory processes that compromise vascular function. The endothelial lining is disrupted, showing signs of cellular apoptosis and irregularities in the vascular structure. There is evidence of endothelial damage, with leukocyte infiltration and inflammatory cell accumulation, particularly in the tunica intima and adventitia. The internal elastic lamina appears fragmented in certain areas, suggesting ongoing vascular remodelling and loss of normal structural integrity. The tunica media, which is critical for vascular tone, exhibits hyperplasia, indicating a response to vascular injury. Smooth muscle cells appear disorganized, and signs of extracellular matrix deposition suggest an attempt at repair, but at the cost of increased stiffness and loss of normal vessel elasticity. Additionally, erythrocyte extravasation into the surrounding tissues suggests increased vascular permeability, a hallmark of endothelial dysfunction in SAH. These pathological features contrast sharply with the control group and establish the baseline for evaluating the potential therapeutic effects of colchicine in mitigating SAH-induced vascular damage. These descriptive features are based on representative images; however, between-group differences in lumen diameter and wall thickness were not statistically significant (p>0.05).

Histopathological changes in the colchicine-treated group are depicted in Figure 3A, demonstrating the vascular response following colchicine administration in the context of SAH. Compared to the untreated SAH group, which typically exhibits severe endothelial damage, inflammatory infiltration, and vascular remodelling, the colchicine-treated basilar artery shows noticeable improvements in structural integrity. The endothelial lining appears more continuous, with a reduction in apoptotic cell bodies and cellular debris. The tunica media, which is often thickened and irregular in SAH conditions, shows less hyperplasia and smoother organization of smooth muscle cells. Importantly, the arterial lumen, which is often constricted in SAH due to vasospasm and inflammatory processes, is more open, suggesting that colchicine treatment may have mitigated some degree of vascular dysfunction. Despite these improvements, residual signs of inflammation and tissue remodelling are still present. While colchicine appears to reduce leukocyte infiltration and oxidative stress markers, small clusters of inflammatory cells can still be observed, particularly in the adventitial region. This suggests that colchicine, while effective in dampening inflammation, does not fully reverse SAH-induced vascular damage. The relative preservation of the extracellular matrix and the reduction of endothelial apoptosis indicate that colchicine’s anti-inflammatory and cytoprotective properties contribute to mitigating SAH-induced vascular injury. However, some degree of vascular remodelling remains evident, highlighting the need for additional interventions to fully restore normal arterial function. Overall, these qualitative improvements did not translate into significant group differences in morphometric measures (p>0.05).

Figure 3B provides a broader view of the overall vascular architecture in the colchicinetreated group at low magnification, allowing for the assessment of both localized and widespread histopathological changes. Compared to the untreated SAH group, the arterial lumen appears less constricted, suggesting that colchicine may play a role in alleviating SAHinduced vasospasm. The thickness of the arterial wall, which is typically increased in response to inflammation and vascular remodelling, is closer to that of the control group, indicating a partial restoration of normal vascular structure. However, some residual thickening is still noticeable, suggesting that colchicine treatment, while beneficial, does not entirely prevent structural changes following SAH. A significant reduction in inflammatory infiltration is evident, particularly in the adventitia, where leukocytes are less densely packed compared to SAH-affected arteries. This attenuation of inflammation is consistent with colchicine’s known inhibitory effects on inflammatory pathways, particularly in reducing cytokine-mediated responses such as TNF-α activity. However, in our study, IL-6 levels were elevated in the colchicine group, which may reflect compensatory upregulation or tissuespecific effects. Despite the reduction in cellular damage, minor signs of hypoxia and vascular stress persist, as indicated by occasional pyknotic nuclei and mild endothelial irregularities. These findings reinforce the therapeutic potential of colchicine in mitigating vascular inflammation and remodelling associated with SAH, although additional therapeutic strategies may be required to achieve full recovery of arterial function. The overall improvement in luminal patency and reduction in inflammatory markers suggests that colchicine contributes to preserving cerebrovascular integrity in SAH conditions, supporting its potential as a neuroprotective agent.

The basilar artery diameter and arterial wall thickness did not differ significantly among groups (p>0.05), so inferences of vascular remodelling from morphology should be considered qualitative.

### 3.2. Biochemical Parameter Analysis

Elevated oxidative stress markers such as TOS and OSI in both tissue and serum, coupled with low TAS, highlight a pronounced oxidative imbalance. This is further supported by histopathological findings, as shown in Figure 2, which illustrates apparent endothelial disruption, inflammatory infiltration, and narrowing of the arterial lumen in the SAH group. In contrast, Figure 1 demonstrates the normal histological structure of the basilar artery in the control group, serving as a baseline for comparison. The higher OSI values in serum compared to tissue suggest systemic oxidative damage extending beyond localized tissue effects (Table 1).

Inflammatory cytokines, including IL-1β, IL-6, and TNF-α, show substantial increases in both compartments, emphasizing a strong inflammatory response likely contributing to vascular and endothelial damage. Notably, TNF-α levels are particularly high in serum, underscoring systemic immune activation. Hypoxia-Inducible Factor-1 Alpha (HIF-1α) and Cytokeratin 18-M65 are also elevated, indicating cellular hypoxia and apoptosis, respectively, further supporting the presence of extensive tissue injury (Tables 2–3). These findings correlate with the extensive endothelial disruption seen in the SAH group (Figure 2).

Thiol-disulfide homeostasis is notably disrupted, with elevated disulfide levels and high Disulfide/Native Thiol ratios in serum, suggesting oxidative protein damage and compromised antioxidant reserves. These findings reinforce the severity of oxidative stress, systemic inflammation, and redox imbalance. In the colchicine-treated group, partial restoration of endothelial structure and reduced inflammation are observed, as seen in Figures 3A and 3B, despite no improvement in serum thiol–disulfide balance or inflammatory cytokines; several serum indices were equal to or higher than SAH. However, residual endothelial damage and arterial wall thickening are still present, indicating incomplete vascular recovery (Figure 3A).

Collectively, the data provide valuable insight into the complex pathophysiological processes triggered by SAH and highlight specific effects: histologic improvement without systemic biochemical attenuation at 48 h (Figures 1, 2, 3A, 3B).

The basilar artery diameter did not show a statistically significant difference (p>0.05) among the Control, SAH, and Colchicine groups. Similarly, arterial wall thickness also demonstrated no significant differences (p>0.05) among these groups. TOS was found to be significantly higher (p<0.05) in both the SAH and Colchicine groups compared to the Control group, while no significant difference (p>0.05) was seen between the SAH and Colchicine groups. The TAS was found to be significantly higher (p<0.05) in the Control group compared to both the SAH and Colchicine groups. However, there was no significant difference (p>0.05) between the SAH and Colchicine groups (Table 2).

The OSI was found to be significantly higher (p<0.05) in both the SAH and Colchicine groups compared to the Control group, while no significant difference (p>0.05) was seen between the SAH and Colchicine groups. IL-1β levels were significantly higher (p<0.05) in both the SAH and Colchicine groups compared to the Control group, while the difference between the SAH and Colchicine groups was not statistically significant (p>0.05). IL-6 levels were found to be significantly higher (p<0.05) in the Colchicine group compared to both the Control and SAH groups. Additionally, the IL-6 level in the SAH group was found to be significantly higher (p<0.05) than in the Control group. TNF-α levels were found to be significantly higher (p<0.05) in both the SAH and Colchicine groups compared to the Control group, while no significant difference (p>0.05) was seen between the SAH and Colchicine groups (Table 2). HIF-1α levels were found to be significantly higher (p<0.05) in both the SAH and Colchicine groups compared to the Control group, with no significant difference (p>0.05) between the SAH and Colchicine groups. Cytokeratin 18-M65 levels were found to be significantly higher (p<0.05) in both the SAH and Colchicine groups compared to the Control group, while no significant difference (p>0.05) was seen between the SAH and Colchicine groups (Table 2).

The TOS was found to be significantly higher (p<0.05) in both the SAH and Colchicine groups compared to the Control group, while no significant difference (p>0.05) was seen between the SAH and Colchicine groups. The TAS was found to be significantly higher (p<0.05) in the Control group compared to both the SAH and Colchicine groups. Additionally, TAS in the SAH group was found to be significantly higher (p<0.05) than in the Colchicine group (Table 3).

The OSI was found to be significantly higher (p<0.05) in the Colchicine group compared to both the Control and SAH groups. OSI was also significantly higher (p<0.05) in the SAH group compared to the Control group. The Total Thiol level was significantly higher (p<0.05) in the Control group compared to both the SAH and Colchicine groups, with no significant difference (p>0.05) between the SAH and Colchicine groups (Table 3).

The Native Thiol level was found to be significantly higher (p<0.05) in the Control group compared to both the SAH and Colchicine groups. Additionally, the SAH group had significantly higher (p<0.05) Native Thiol levels than the Colchicine group. Disulfide levels were found to be significantly higher (p<0.05) in both the SAH and Colchicine groups compared to the Control group, while no significant difference (p>0.05) was seen between the SAH and Colchicine groups. The percentage of Native Thiol/Total Thiol was found to be significantly higher (p<0.05) in the Control group compared to both the SAH and Colchicine groups. Moreover, this percentage was significantly higher (p<0.05) in the SAH group compared to the Colchicine group. The percentage of Disulfide/Total Thiol was found to be significantly higher (p<0.05) in the Colchicine group compared to both the Control and SAH groups, and significantly higher (p<0.05) in the SAH group compared to the Control group (Table 3).

The percentage of Disulfide/Native Thiol was found to be significantly higher (p<0.05) in the Colchicine group compared to both the Control and SAH groups. It was also significantly higher in SAH than Control (p<0.05). IL-1β levels were found to be significantly higher (p<0.05) in the Colchicine group compared to both the Control and SAH groups, while the SAH group also had significantly higher (p<0.05) IL-1β levels than the Control group. IL-6 levels were found to be significantly higher (p<0.05) in the Colchicine group compared to both the Control and SAH groups, while the SAH group also exhibited significantly higher (p<0.05) IL-6 levels compared to the Control group. TNF-α exhibited significantly higher levels (p<0.05) in the Colchicine group compared to both the Control and SAH groups. TNFα levels in the SAH group were also significantly higher (p<0.05) than in the Control group (Table 3).

HIF-1α levels were found to be significantly higher (p<0.05) in both the SAH and Colchicine groups compared to the Control group, while no significant difference (p>0.05) was seen between the SAH and Colchicine groups. Cytokeratin 18-M65 levels were found to be significantly higher (p<0.05) in the Colchicine group compared to both the Control and SAH groups. The SAH group also showed significantly higher (p<0.05) levels of Cytokeratin 18-M65 compared to the Control group (Table 3).

## 4. Discussion

Our study found significantly elevated levels of pro-inflammatory cytokines, including IL-1β, IL-6, and TNF-α, in the SAH group, which correlated with extensive vascular damage as shown in Figures 1 and 2. These findings are consistent with previous reports linking cytokine overproduction to cerebral vasospasm and endothelial injury. Colchicine was associated with partial histological improvement; however, it did not attenuate systemic cytokines or oxidative stress indices, and structural vascular parameters such as basilar artery diameter and wall thickness remained unchanged.

The dissociation between partial histological improvement and persistent biochemical deterioration may be attributed to several factors. First, the 48-hour observation period may be insufficient to capture morphological changes that require longer time frames to develop or reverse. Inflammatory cascades can be rapidly modulated by agents such as colchicine, whereas endothelial regeneration and vascular remodelling often occur over longer durations. Previous reports have highlighted the time-dependent nature of SAH-induced vascular damage and the role of persistent inflammation in driving vasospasm and arterial wall changes [3, 5].

Second, colchicine’s mechanisms may explain its inability to affect vascular diameter or wall thickness acutely. Although colchicine inhibits neutrophil infiltration and reduces inflammasome activation [12], its primary actions target microtubule dynamics and cytokine signalling rather than direct vascular remodelling. It has shown beneficial effects in cardiovascular diseases, including myocardial infarction and percutaneous coronary intervention, largely through modulation of systemic inflammation and prevention of leukocyte-endothelial adhesion [11, 13, 16, 17]. However, its specific impact on cerebral vascular endothelial cells and smooth muscle after haemorrhagic events remains poorly characterized.

These findings emphasize the complex, multifactorial pathophysiology of SAH-related vascular injury, involving oxidative stress, inflammation, apoptosis, and impaired vascular tone [3, 5, 7]. Targeting inflammation alone may not suffice for complete vascular protection. Future approaches may require combination strategies incorporating anti-inflammatory agents like colchicine along with vasodilators or endothelial regenerative therapies.

From a translational standpoint, colchicine’s favourable safety profile and low cost support its candidacy for adjunctive use in neurovascular disorders. However, further studies should investigate its long-term vascular effects, optimal timing, and potential synergy with other agents. Functional outcomes and extended histopathological analyses at later time points would enhance understanding of its therapeutic potential in SAH.

In this context, previous experimental work with vitamin B12 in a similar rat SAH model has demonstrated improvements in endothelial integrity and reductions in oxidative stress, as shown by microscopic, stereological, and biochemical analyses. While vitamin B12 primarily exerts antioxidant and neuroprotective effects and colchicine acts mainly through anti-inflammatory pathways, both target key mechanisms of SAH-induced vascular injury [22]. These findings support the rationale for multi-targeted therapeutic approaches combining anti-inflammatory and antioxidant strategies for more comprehensive cerebrovascular protection.

The current study showed pronounced oxidative and inflammatory activation after SAH and only partial, qualitative histological improvement with colchicine, without concomitant biochemical benefit at 48 hours. We observed pronounced oxidative and inflammatory responses after SAH and modest endothelial improvement with colchicine on histology, but systemic biochemical markers did not improve at the study time point.

Our results showed a marked increase in TOS and OSI in the SAH group compared to the control group, confirming the role of oxidative stress in SAH pathophysiology [3, 8] (Table 2 and Table 3, Figures 1, 2). Oxidative damage after SAH is primarily mediated by free radicals such as reactive oxygen species (ROS), which cause lipid peroxidation, protein denaturation, and DNA damage. While histological analyses demonstrated modest endothelial restoration (Figures 3A–B), serum oxidative markers (e.g., OSI) were not reduced and were frequently higher than those observed in the SAH group (Table 3). This suggests that colchicine may alleviate oxidative injury by indirectly modulating the inflammatory response and ROS production through its inhibition of neutrophil activation and microtubule stabilization. [9, 12].

Interestingly, the TAS was significantly lower in the SAH and Colchicine groups compared to the control group, indicating persistent oxidative imbalance despite treatment (Table 2). This result highlights the need for combining colchicine with direct antioxidant therapies for more comprehensive management.

The inflammatory response plays a pivotal role in SAH-induced vasospasm and secondary brain injury [3, 5, 7]. Our study found significantly elevated levels of pro-inflammatory cytokines such as IL-1β, IL-6, and TNF-α in the SAH group correlated with extensive vascular damage, as shown in Figures 1 and 2 consistent with prior reports linking cytokine overproduction to cerebral vasospasm and endothelial damage [5, 6]. Colchicine did not reduce IL-1β or TNF-α; in serum these cytokines were higher than SAH (Table 3), and tissue patterns also favoured higher values vs. Control (Table 2). Taken together, these findings indicate that under the dosing and timing applied in this study, systemic cytokine suppression and oxidative stress attenuation were not achieved. Notably, IL-6 levels were paradoxically elevated in the colchicine group, suggesting differential regulation or a compensatory feedback mechanism.

Histopathological analysis revealed no significant differences in basilar artery diameter and arterial wall thickness across groups, as shown in Figures 1 and 2, suggesting that colchicine did not directly affect structural vascular remodelling. However, markers of vascular damage, including HIF-1α and Cytokeratin 18-M65, were significantly elevated in the SAH and Colchicine groups (Table 3, Figures 2, 3A). This indicates persistent endothelial injury despite histologic improvement, likely due to irreversible damage from the initial haemorrhagic event [5, 13].

Additionally, thiol-disulfide homeostasis analysis demonstrated reduced Total Thiol and Native Thiol levels and elevated Disulfide levels in the SAH and Colchicine groups (Table 2), reflecting oxidative protein damage and antioxidant depletion. These findings align with the observed histopathological changes, as seen in Figures 1 and 2, and suggest the need for comprehensive treatment strategies targeting redox balance alongside inflammation control [8, 14].

Our findings align with previous studies highlighting colchicine’s anti-inflammatory potential in cardiovascular diseases, including myocarditis and post-infarction management [13, 14]. While prior research has focused on cardiac protection, our study extends these findings to cerebrovascular pathology, suggesting that colchicine may mitigate SAH-induced vascular damage through similar mechanisms.

However, the lack of complete recovery in oxidative and vascular markers underscores the complexity of SAH pathology and suggests that colchicine alone may be insufficient for complete vascular protection (Figures 3A, 3B). Combination therapies involving antioxidants, vasodilators, and neuroprotective agents could enhance therapeutic efficacy, as histopathological evidence.

## 5. Conclusion

Colchicine produced partial histological improvement without parallel reductions in systemic oxidative stress or cytokines at the study time point and did not induce significant structural recovery in the acute phase after SAH. These results provide a foundation for further investigation into multimodal therapies targeting both biochemical and morphological aspects of SAH-induced vascular injury.

Overall, colchicine was associated with partial histological improvement without systemic biochemical benefit at the study time point. Notably, IL-6 levels were elevated in the colchicine group, which may reflect a compensatory or pathway-specific cytokine response. Likewise, colchicine did not lower IL-1β or TNF-α; in fact, these cytokines were increased relative to SAH in serum, and tissue measurements also tended toward higher values compared with Control (Tables 2–3); future work should test alternate dosing/timing and larger cohorts.

Overall, colchicine provided limited histological benefit without systemic biochemical improvement, underscoring the need for further studies with alternative dosing strategies, extended time points, and larger cohorts to clarify its therapeutic potential.

## 6. Limitations

This study has several limitations. The relatively small sample size may restrict the generalizability of findings. Additionally, the fixed-dose regimen of colchicine limits insights into dose-dependent effects, necessitating exploration of varying doses and treatment intervals for better understanding. The short observation period (48 hours post-SAH) also limits the ability to evaluate long-term vascular and biochemical changes. Lastly, while this study focused on key oxidative stress and inflammatory markers, broader biochemical and histopathological analyses would provide more comprehensive insights into colchicine’s cerebrovascular effects.

## 7. Suggestions for Further Research

1. Combination therapies: Testing colchicine with direct antioxidants, vasodilators, and neuroprotective agents.
2. Dosage and timing optimization: Investigating dose-response relationships, timing of treatment initiation, and therapy duration.
3. Mechanistic insights: Exploring molecular pathways of colchicine’s action in cerebrovascular inflammation and oxidative stress.
4. Clinical trials: Translating preclinical findings into clinical settings to assess the feasibility of colchicine as an adjunct treatment for SAH.

Colchicine showed partial histological improvement but did not attenuate systemic cytokines or oxidative stress indices in the study timeline. However, its limited effects on oxidative imbalance and vascular structure highlight the need for multimodal approaches. Future research focusing on combination therapies and mechanistic insights will be crucial in developing effective cerebrovascular protection strategies.

## Figures and Tables

**Figure 1:**
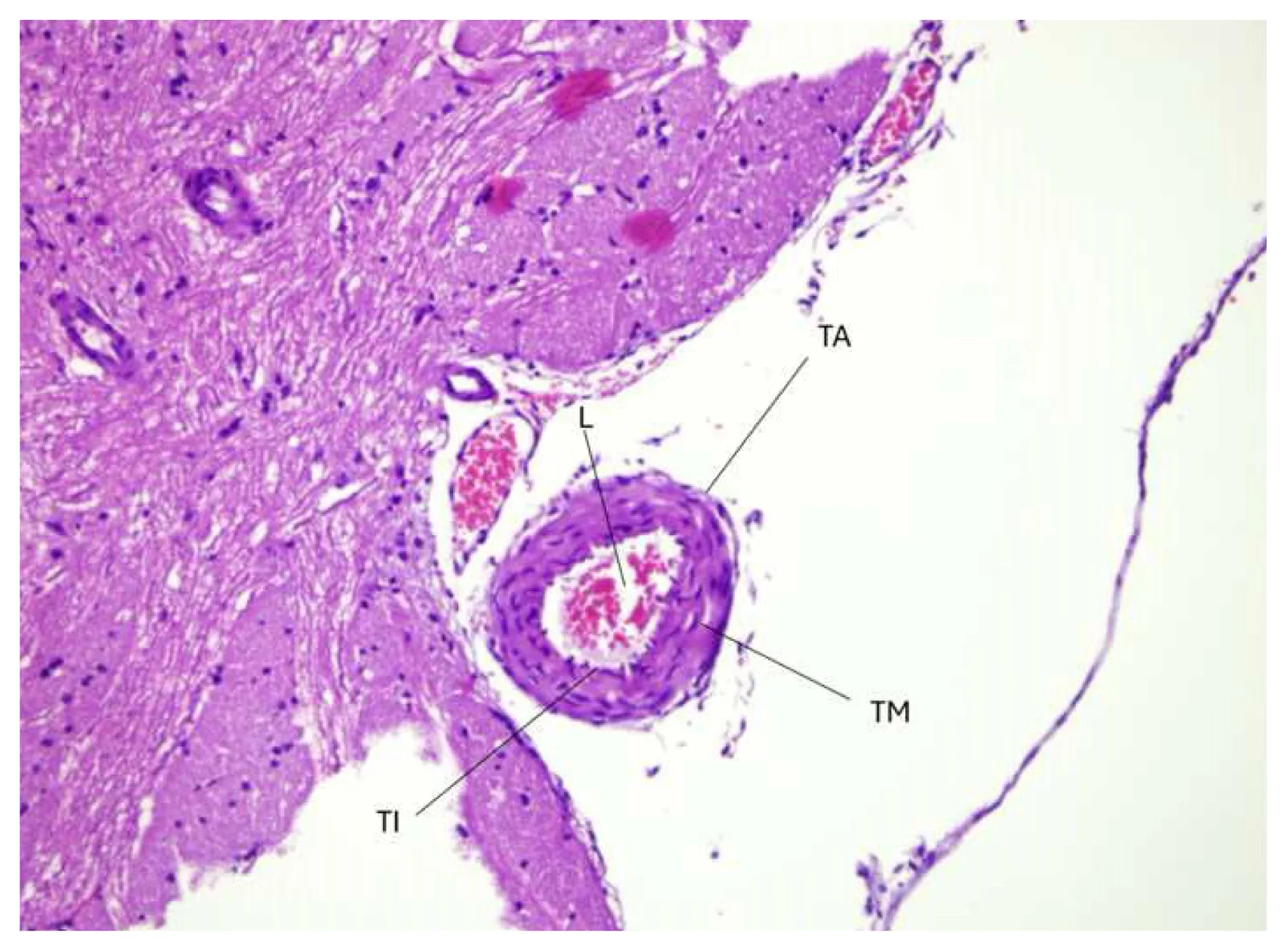
Light microscopy findings (H&E staining, 200×). A medium-sized artery showing tunica intima (TI), tunica media (TM), and tunica adventitia (TA) layers with an intact endothelial lining and smooth muscle cell organization. The lumen (L) contains erythrocytes. Surrounding connective tissue and adjacent smaller vessels are visible. Scale bar: 50 μm.

**Figure 2:**
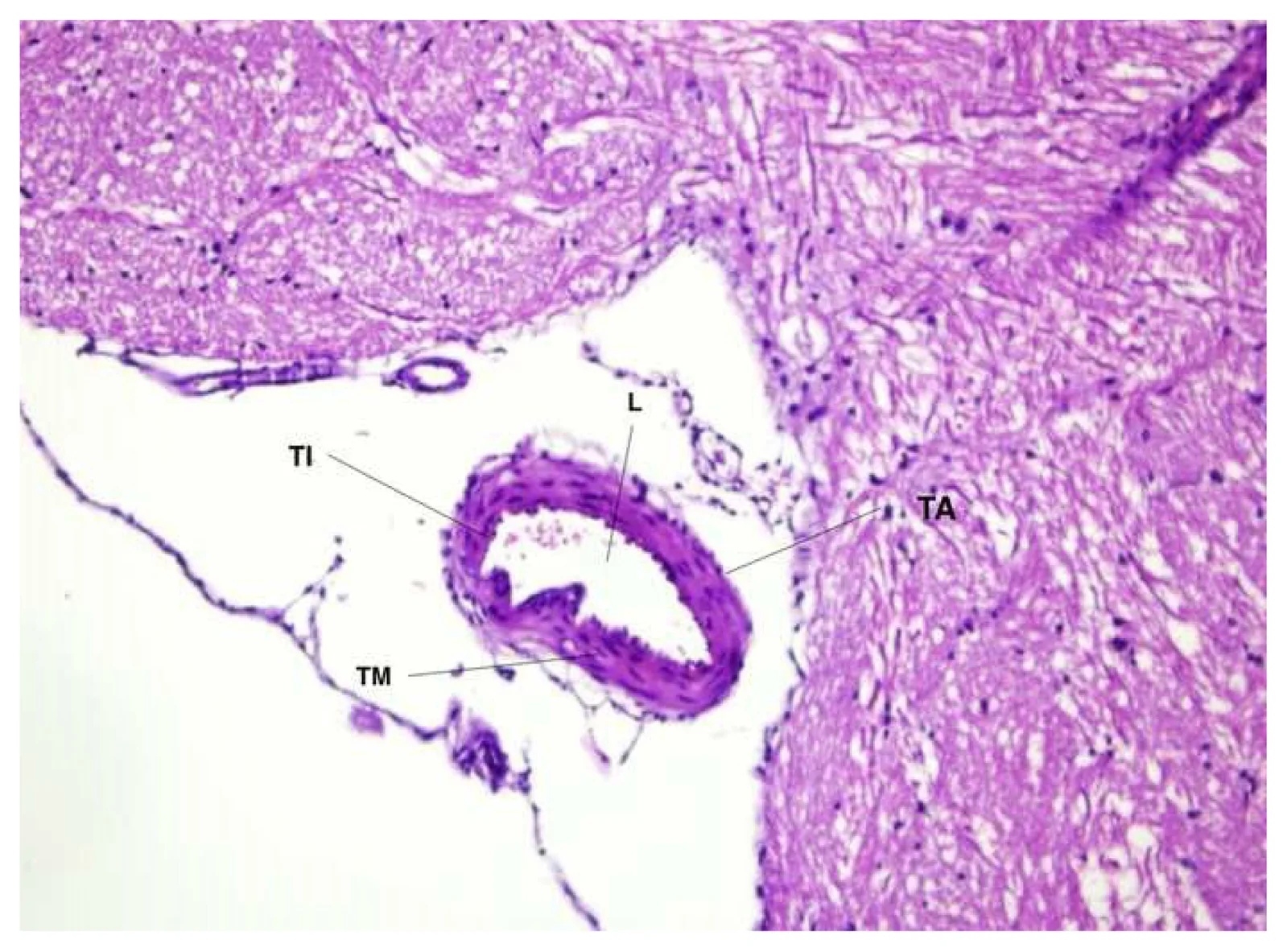
Light microscopy findings (H&E staining, 200×). An arterial section with representative features suggestive of wall thickening with narrowing of the lumen (L). The tunica media (TM) appears hypertrophic, and the tunica intima (TI) is thickened with vascular remodeling. Surrounding tissue shows preserved parenchymal architecture. Scale bar: 50 μm.

**Figure 3:**
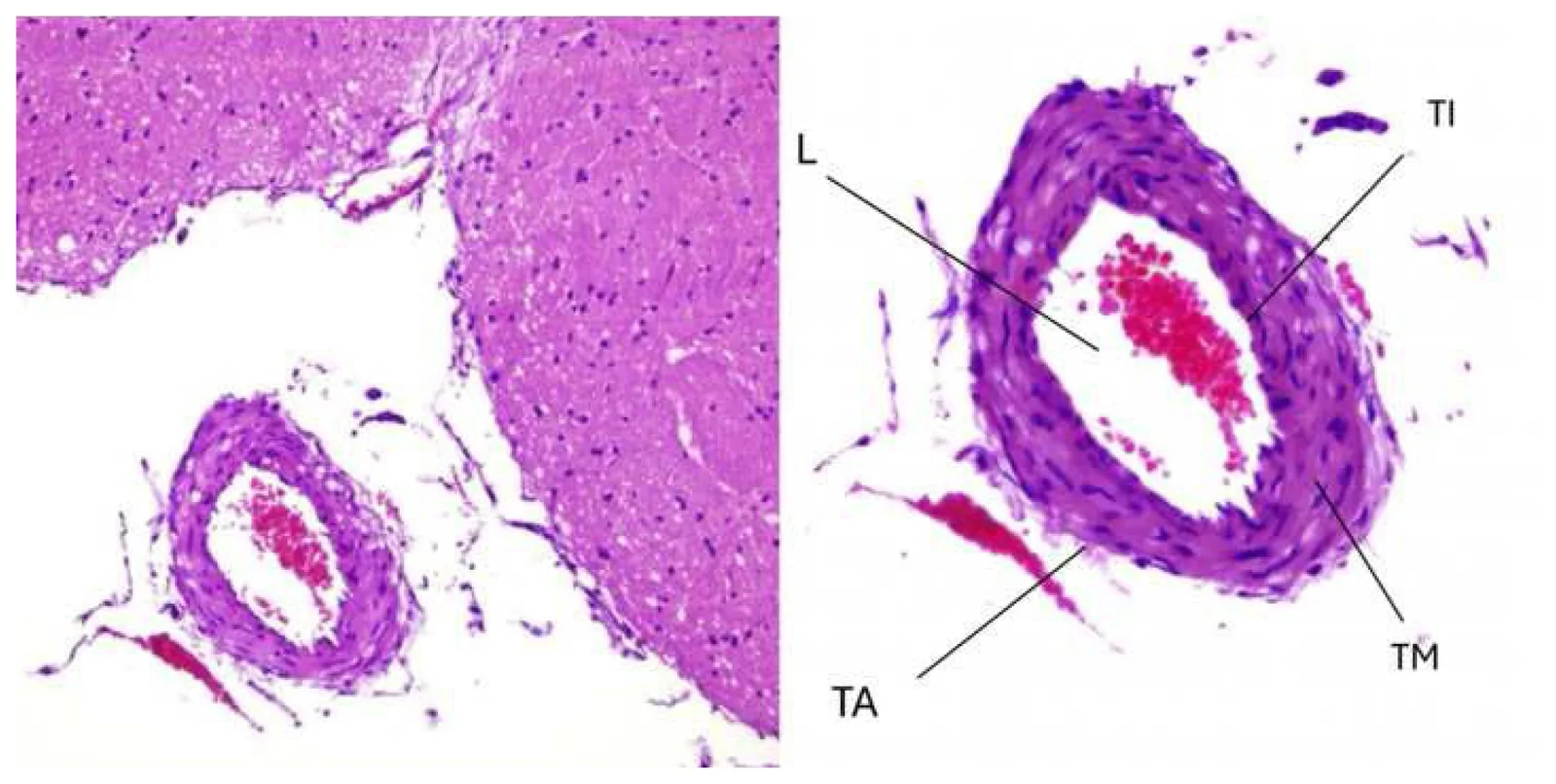
Light microscopy findings (H&E staining, 200×). A) Arterial section showing tunica intima (TI), tunica media (TM), and tunica adventitia (TA) layers with preserved organization. The lumen (L) contains erythrocytes, and the surrounding parenchymal tissue exhibits intact morphology. B) Higher magnification view of the artery in panel A, highlighting the organization of the tunica intima and tunica media. Scale bar: 50 μm.

**Table 1:** Descriptive statistics of tissue and serum biochemical markers.

Parameter (with Unit)	Min – Max	Mean ± SD	Sample
Basilar Artery Diameter (μm)	89.3 – 226.8	136.1 ± 34.8	Tissue
Arterial Wall Thickness (μm)	24.5 – 56.4	38.1 ± 8.8	Tissue
Total Oxidant Status (μmol H_2_O_2_ eq/L·mg protein)	11.2 – 2.8	6.2 ± 2.7	Tissue
Total Antioxidant Status (mmol AA eq/L·mg protein)	0.21 – 0.91	0.54 ± 0.21	Tissue
Oxidative Stress Index (AU)	3.8 – 38.1	15.3 ± 11.0	Tissue
Interleukin-1 Beta (ng/L·mg protein)	4.4 – 12.3	8.9 ± 2.5	Tissue
Interleukin-6 (ng/L·mg protein)	1.1 – 5.6	3.8 ± 1.6	Tissue
Tumor Necrosis Factor Alpha (ng/L·mg protein)	37.3 – 161.9	104.8 ± 40.8	Tissue
Hypoxia Inducible Factor 1 Alpha (ng/ml·mg protein)	0.31 – 1.13	0.82 ± 0.27	Tissue
Cytokeratin 18-M65 (ng/L·mg protein)	110.3 – 563.8	303.9 ± 133.1	Tissue
Total Oxidant Status (μmol H_2_O_2_ eq/L·mg protein)	3.9 – 12.2	8.4 ± 2.5	Serum
Total Antioxidant Status (mmol AA eq/L·mg protein)	0.15 – 1.17	0.63 ± 0.35	Serum
Oxidative Stress Index (AU)	3.5 – 73.7	20.8 ± 16.8	Serum
Total Thiol (μM)	223.8 – 569.1	331.5 ± 111.5	Serum
Native Thiol (μM)	14.1 – 393.4	155.3 ± 149.9	Serum
Disulfide (μM)	40.9 – 141.7	88.1 ± 29.1	Serum
% Native Thiol / Total Thiol (%)	6.0 – 81.4	38.9 ± 28.8	Serum
% Disulfide / Total Thiol (%)	9.3 – 47.0	30.5 ± 14.4	Serum
% Disulfide / Native Thiol (%)	11.4 – 786.0	200.2 ± 218.5	Serum
Interleukin-1 Beta (ng/L·mg protein)	7.3 – 40.0	20.7 ± 10.3	Serum
Interleukin-6 (ng/L·mg protein)	5.4 – 34.5	22.2 ± 10.7	Serum
Tumor Necrosis Factor Alpha (ng/L·mg protein)	91.8 – 538.4	303.1 ± 146.5	Serum
Hypoxia Inducible Factor 1 Alpha (ng/ml·mg protein)	1.5 – 5.8	3.8 ± 1.6	Serum
Cytokeratin 18-M65 (ng/L·mg protein)	123.7 – 616.6	340.9 ± 157.8	Serum

**Table 2:** Comparative analysis of biochemical and histopathological parameters among control, SAH, and colchicine groups.

Parameter	Control G. Mean ± SD	SAH G. Mean ± SD	Colchicine G. Mean ± SD	p	Test
Basilar Artery Diameter (μm)	153.4 ± 47.3	128.3 ± 35.8	126.8 ± 7.4	=0.459	K
Arterial Wall Thickness (μm)	39.9 ± 13.3	37.3 ± 5.0	37.1 ± 7.6	=0.847	A
Total Oxidant Status	3.1 ± 0.2	7.9 ± 1.3	7.7 ± 2.4	<0.001	A
Total Antioxidant Status	0.79 ± 0.07	0.45 ± 0.19	0.38 ± 0.03	<0.001	A
Oxidative Stress Index	3.9 ± 0.2	21.3 ± 11.0	20.6 ± 7.8	=0.003	K
Interleukin-1 Beta	5.8 ± 1.0	9.8 ± 0.8	11.0 ± 0.8	<0.001	A
Interleukin-6 (ng/L·mg)	1.7 ± 0.3	4.6 ± 0.3	5.2 ± 0.3	<0.001	A
TNF-α (ng/L·mg)	52.3 ± 10.5	123.2 ± 13.8	138.9 ± 15.6	<0.001	A
HIF-1α (ng/ml·mg)	0.47 ± 0.11	0.98 ± 0.05	1.01 ± 0.11	<0.001	A
Cytokeratin 18-M65	153.7 ± 36.5	349.3 ± 80.7	408.7 ± 98.5	<0.001	A

^K^ Kruskal-wallis (Mann-whitney u test) / ^A^ ANOVA (Tukey test)

Difference With ^1^Control Group p<0.05;

Difference With ^2^SAH Group p<0.05

Difference With ^3^ Colchicine Group p<0.05

**Table 3:** Comparative analysis of serum biochemical and inflammatory markers among control, SAH, and colchicine groups.

Parameter	Control G. Mean ± SD	SAH G. Mean ± SD	Colchicine G. Mean ± SD	p	Test
Total Oxidant Status	5.4 ± 1.7	9.6 ± 0.6	10.2 ± 1.4	<0.001	A
Total Antioxidant Status	1.09 ± 0.08	0.47 ± 0.05	0.31 ± 0.10	<0.001	A
Oxidative Stress Index	5.0 ± 1.5	20.4 ± 2.8	36.9 ± 18.3	=0.001	K
Total Thiol (μM)	472.9 ± 49.1	284.4 ± 49.6	237.3 ± 6.0	=0.001	K
Native Thiol (μM)	357.2 ± 26.8	83.0 ± 17.2	25.8 ± 8.5	=0.001	K
Disulfide (μM)	57.9 ± 20.7	100.7 ± 27.5	105.7 ± 5.5	=0.002	A
% Native Thiol / Total Thiol	75.9 ± 6.7	30.0 ± 8.6	10.9 ± 3.6	<0.001	A
% Disulfide / Total Thiol	12.0 ± 3.3	35.0 ± 4.3	44.6 ± 1.8	<0.001	A
% Disulfide / Native Thiol	16.3 ± 6.2	126.5 ± 42.7	457.8 ± 183.8	<0.001	A
Interleukin-1 Beta	9.2 ± 2.1	21.5 ± 5.4	31.4 ± 5.2	=0.001	K
Interleukin-6 (ng/L·mg)	7.9 ± 1.5	27.5 ± 2.1	31.1 ± 2.8	<0.001	A
TNF-α (ng/L·mg)	117.4 ± 21.5	349.2 ± 52.0	442.7 ± 49.8	=0.001	K
HIF-1α (ng/ml·mg)	1.6 ± 0.2	4.7 ± 0.7	4.9 ± 0.7	<0.001	A
Cytokeratin 18-M65	138.3 ± 8.6	401.1 ± 38.9	483.1 ± 72.1	<0.001	A

^K^ Kruskal-wallis (Mann-whitney u test) / ^A^ ANOVA (Tukey test)

Difference With ^1^Control Group p<0.05;

Difference With ^2^SAH Group p<0.05

Difference With ^3^ Colchicine Group p<0.05
